# Acute Volvulus of the Cecum

**DOI:** 10.5334/jbsr.2112

**Published:** 2020-07-03

**Authors:** Quentin Jacquemin, Bruno Coulier, Raphael Rubay

**Affiliations:** 1 Clinique St Luc, BE; 2 Clinique Saint-Luc, Bouge, BE

**Keywords:** cecal volvulus, mobile cecum, bowel obstruction, abdominal CT

## Abstract

**Teaching Point:** Abnormal embryological cecal fixation may lead to volvulus, which accounts for approximately 1 to 3 percent of all colic obstructions. A pack of suggestive CT features can make the diagnosis.

## Case Report

An 83-year-old woman presented with a six-day history of diffuse abdominal pain, vomiting, and nausea. Physical examination showed abdominal distention and defense without rebound. Abdominal contrast-enhanced computed tomography (CT) was performed.

**Figure 1:**
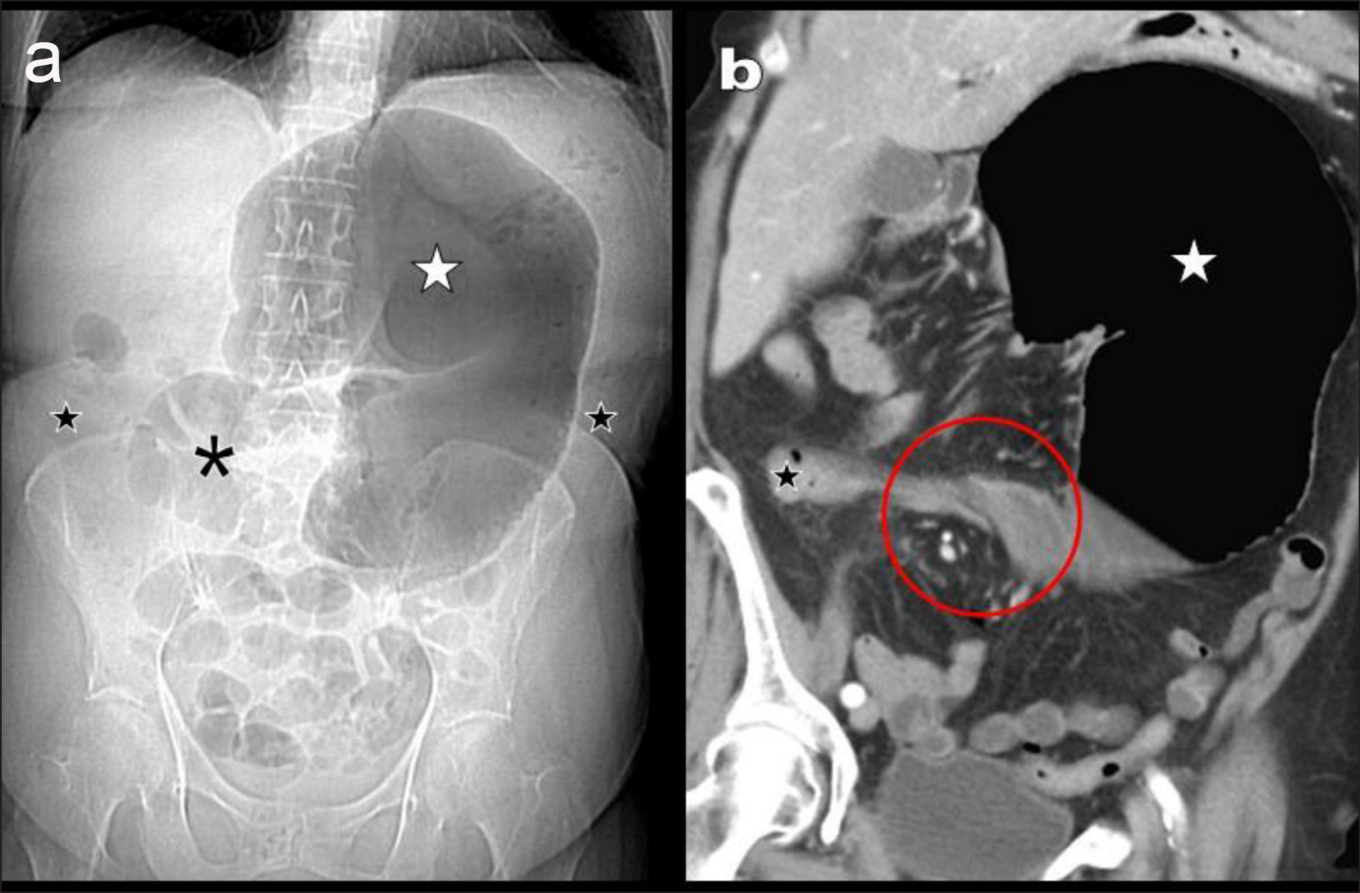


**Figure 2:**
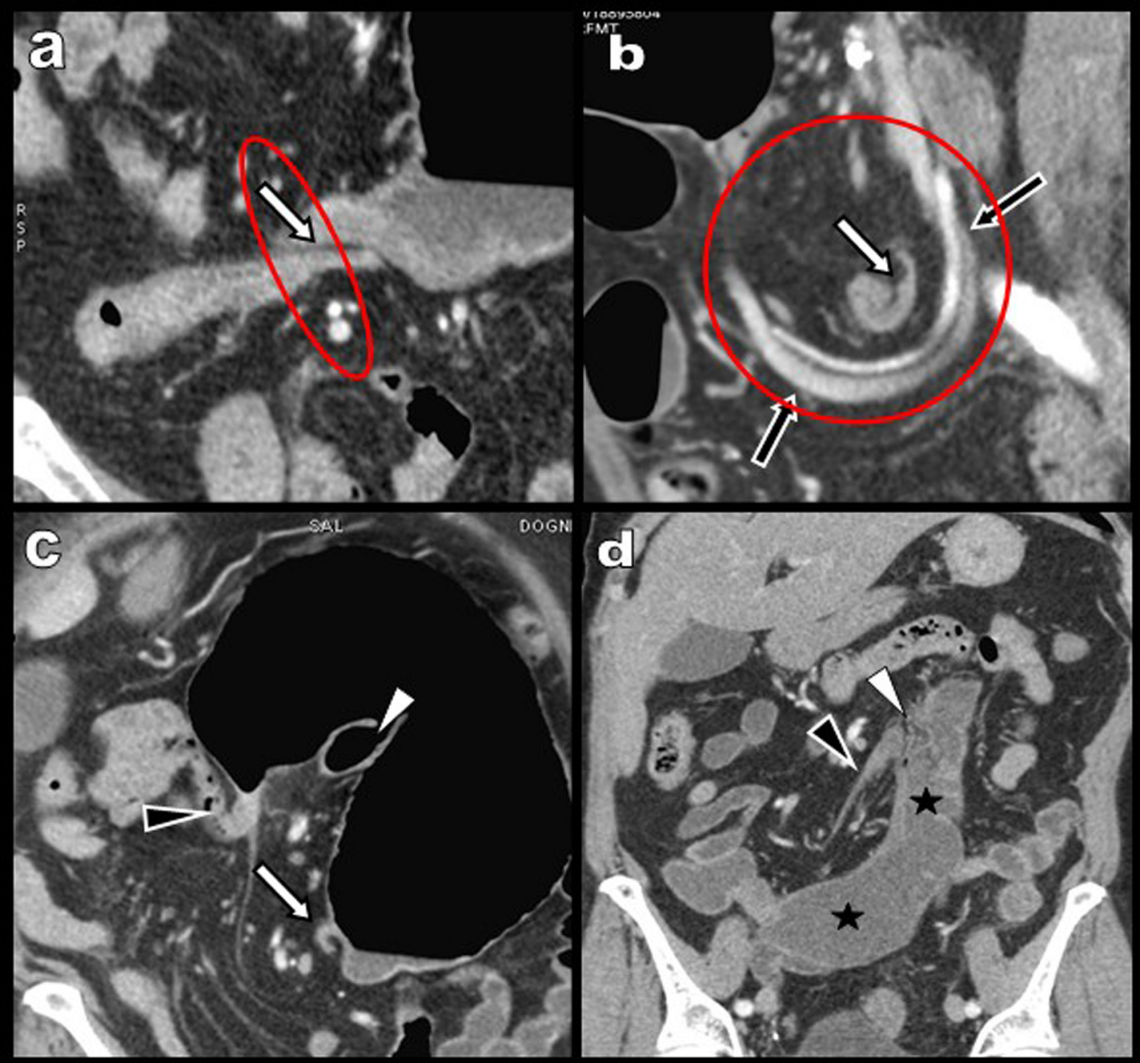


Scout view (Figure 1a) and coronal reformation (Figure 1b) reveal massive air distention of a mainly left-sided “kidney-shaped” part of the large bowel (white star on Figure 1a and 1b). Distal small bowel air distention is also noted (black asterisk). The distal colon appears empty and flat (black stars on Figure 1a and 1b). Thin-slices reformations (Figure 2) identify a “whirl sign” (red circle) proximal to the distended colonic segment identified as the cecum (white star). Complementary signs of volvulus include: (I) a winding of the ileocecal vessels (black arrows on Figure 2) around the twisted cecal neck with a typical “split wall” sign (white arrows on Figure 2a and 2b), (II) a 180° flip of the orientation of the valve of Bauhin at the concave verge of the “kidney shape” cecum (white arrowhead), (III) a flip of the appendix orientation (black arrowhead) and (IV) an inverted orientation of the cecal apex towards the left upper quadrant.

**Figure 3:**
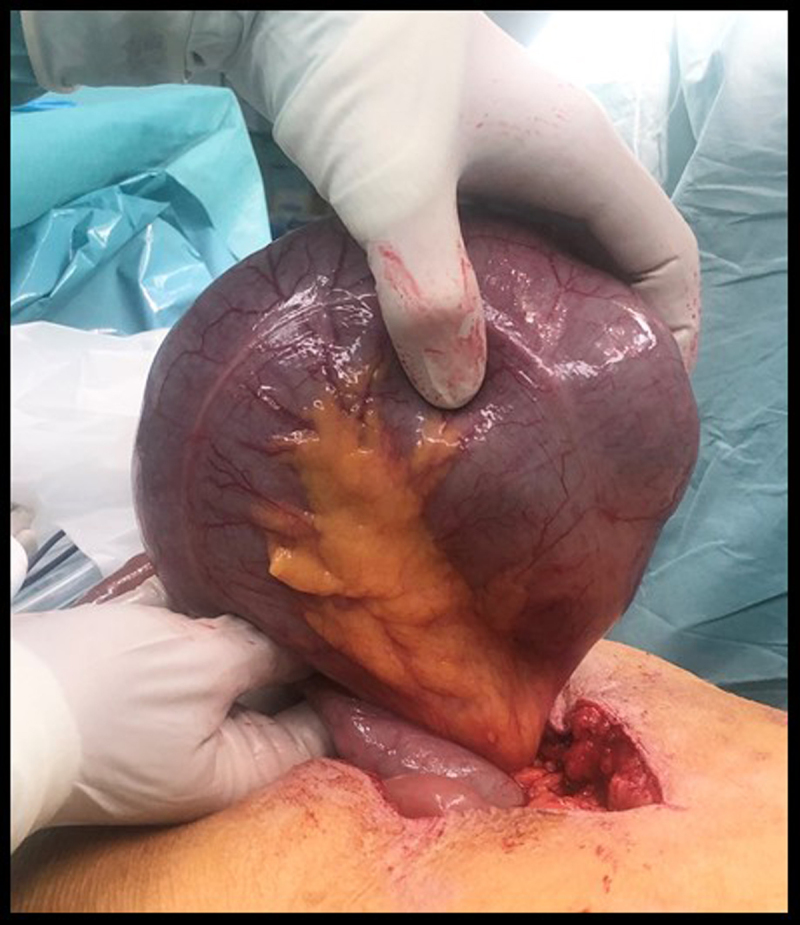


The diagnosis of cecal volvulus is confirmed surgically (Figure 3) and right hemicolectomy is performed with uneventful post-operative recovery. Interestingly, a review of the patient’s imaging data retrieves an abdominal CT performed five years earlier, already showing a wandering long ectopic cecum with neither distention nor volvulus (Figure 2d, black stars).

## Comment

A mobile cecum is anatomically defined as an anomalous position of the right colon, cecum, and terminal ileum due to the failure of the right colon mesentery to fuse with the posterior parietal peritoneum. It is present in 10–20% of the population.

This absence of fixation abnormality allows an abnormal intra-abdominal mobility of the ascending colon and cecum, with a potentially wide range displacement within the abdominal cavity.

Clinical manifestations may include the chronic “mobile cecum syndrome” associating recurrent abdominal pain and constipation and rarely, acute cecal volvulus. The occurrence of the recurrent pattern is reported in about 50% of the patients before the onset of acute volvulus.

Cecal volvulus may be sometimes diagnosed on abdominal plain radiographs. Nevertheless, computed tomography (CT) represents the gold standard. Severe (>10 cm) cecal distention, “coffee bean” sign (or “kidney-shaped” appearance), cecal apex projecting in the left upper quadrant, distal colonic flattening, proximal small bowel distention, whirl sign, ileocecal twist with transition points (beak signs) and split-wall sign are the most commonly reported signs [1].
